# The quiet reservoir: Understanding Shiga toxin *Escherichia coli* O157:H7 colonization in cattle

**DOI:** 10.1371/journal.ppat.1014565

**Published:** 2026-09-10

**Authors:** Katherine P. Feldmann, Phillip R. Myer

**Affiliations:** Department of Animal Science, University of Tennessee, Knoxville, Tennessee, United States of America; Duke University School of Medicine, UNITED STATES OF AMERICA

In the United States, Shiga toxin-producing *Escherichia coli* (STEC) remains a major food safety concern, causing an estimated 357,000 illnesses annually with O157:H7 representing the most clinically significant serovar [1]. Cattle are the primary reservoir of O157:H7, and approximately 20% of illnesses are linked to beef [2]. Consumption of contaminated beef products can cause abdominal cramps, bloody diarrhea, and in severe cases progress to Hemolytic Uremic Syndrome (HUS), a life-threatening condition that can result in acute kidney failure and death [3]. Because of these severe health outcomes, O157:H7 along with six other non-O157 serogroups are classified as adulterants with a zero-tolerance policy in beef products [4]. While other non-O157 STEC serogroups also contribute to human disease, O157:H7 remains the most studied serogroup in cattle. The significance of this foodborne pathogen has driven extensive efforts to understand how O157:H7 colonizes cattle and persists within the bovine gastrointestinal tract (GIT) (Fig 1).

**Fig 1:**
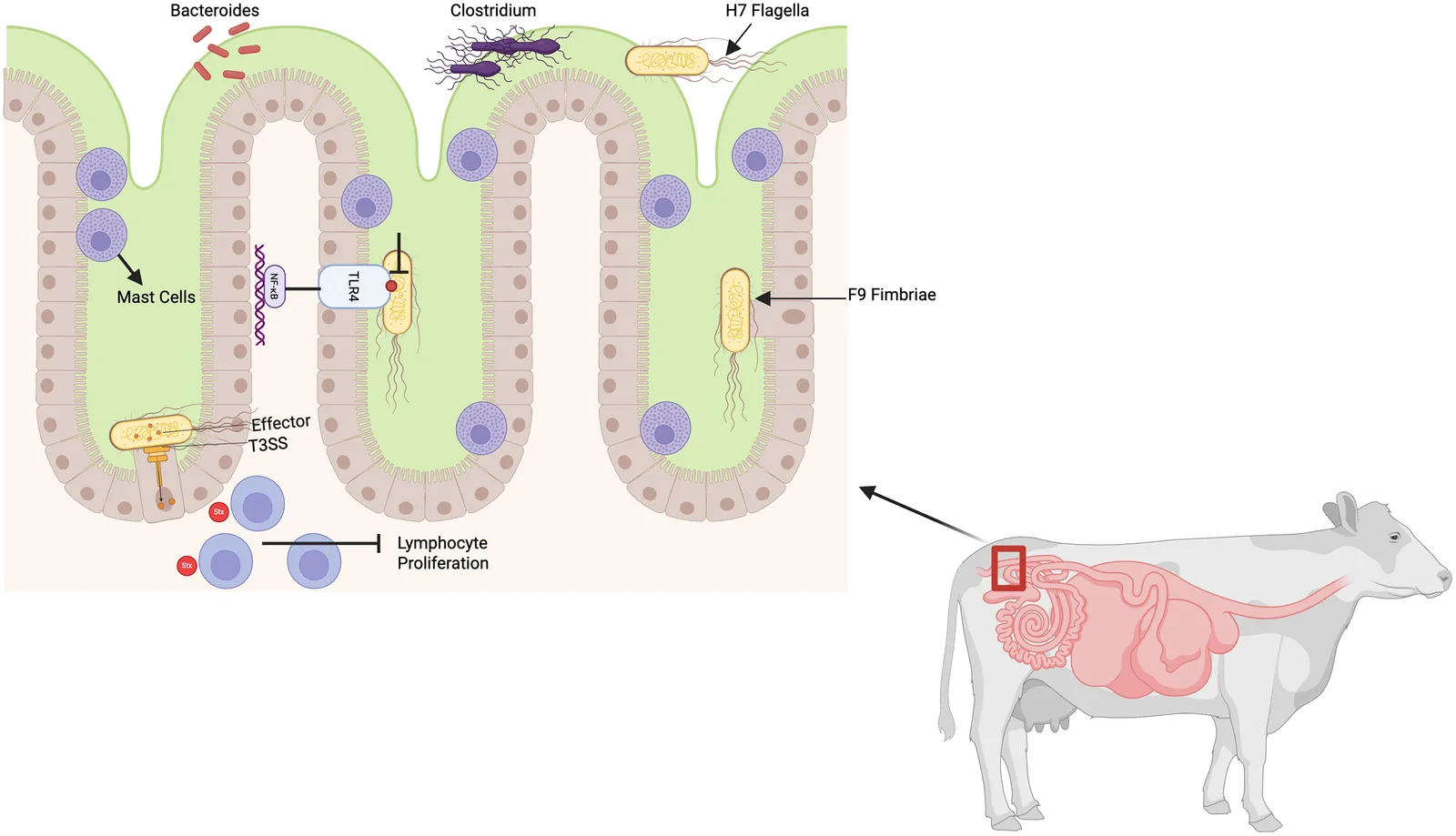
A depiction for what is known for O157:H7 colonization at the RAJ (site highlighted by red box). H7 flagella contribute to enhanced motility, and F9 fimbriae facilitate initial attachment. The secretion of effector proteins from the T3SS injectosome creates attachment and effacement lesions on epithelial cells. Shiga toxin (Stx) interacts with lymphocytes to prevent proliferation. Other effector proteins impact TLR4 signaling and NF-kB activation of proinflammatory factors. Increased mast cell abundance is observed in O157:H7. Additionally, positively correlated bacteria such as *Bacteroides* and *Clostridium* are present. Created in BioRender. Feldmann, **K.** (2026) https://BioRender.com/x1gha8r.

## Why are cattle the primary reservoir for O157:H7?

The infamy of O157:H7 outbreaks associated with beef products, such as the 1993 Jack-in-the-Box outbreak linked to contaminated hamburgers, has positioned cattle as the primary reservoir [5]. How cattle initially become exposed to O157:H7 remains an ongoing research question. Several bird species, including pigeons and starlings, have been identified as carriers of O157:H7, leading to the theory that migratory birds may contribute to the dissemination of new strains between cattle operations [6]. Once introduced, cattle asymptomatically carry O157:H7 within the GIT, enabling continued shedding and environmental transmission. Unlike humans, cattle do not develop hemorrhagic disease following colonization. Cattle gastrointestinal vascular endothelial cells lack globotriaosylceramide (Gb3/CD77), the receptor for Shiga toxin, a primary virulence factor [7]. Consequently, cattle do not exhibit the vascular damage associated with this toxin in human infections. Colonization occurs across all ages and production systems, including feedlot and pasture-based operations. Cattle exhibit highly variable levels of O157:H7 shedding driven by factors such as seasonality, weather, diet, and transport stress. In some feedlot studies, up to 80 percent of animals within a pen were actively shedding O157:H7 [8]. Furthermore, cattle can exhibit a phenomenon known as super shedding, in which animals shed ≥10^4^ CFU/g in feces [9]. These animals disproportionately contribute to environmental contamination and transmission within pen groups, making them a significant concern for food safety.

## How does O157:H7 establish colonization in cattle?

O157:H7 possesses several adaptations that allow it to survive transit through the bovine GIT and establish colonization. Within the rumen, O157:H7 upregulates the arginine-dependent acid resistance system along with additional stress-response genes that promote survival [10]. To withstand the acidic conditions of the abomasum (the true stomach), O157:H7 utilizes the glutamate-dependent acid resistance system encoded by *gadABC* [3]. No single mechanism has been attributed to bile salt resistance in the small intestine. Instead, survival likely depends on multiple stress-responsive pathways. Following transit through the upper GIT, O157:H7 preferentially colonizes the recto-anal junction (RAJ) [11]. The RAJ represents a transition zone from stratified squamous epithelium to columnar epithelial mucosa and submucosal lymphoid follicles. Although the precise reason for O157:H7 tropism toward this site remains unclear, the RAJ likely provides an ideal environment for bacterial adherence and persistence. Several adherence factors contribute to O157:H7 colonization at the RAJ. Cell surface proteins, T5eSS, Intimin and Tir effector together with the T3aSS EscF-EspABD (the molecular structure encoded by the LEE4 operon forming the injectosome), facilitate the formation of attaching and effacing lesions on epithelial cells [3]. These lesions promote intimate attachment and microcolony formation at the RAJ mucosa. Additional motility and adherence factors also support colonization [3]. Flagella, including H7, enhance bacterial motility and promote initial contact with the RAJ epithelium [12]. Fimbriae 9 (F9) has similarly been shown to facilitate adhesion at the RAJ. Additional surface structures, including pili, curli, and other adhesins, likely contribute to colonization, although their precise roles remain unclear.

## How does the bovine GIT microbiota play a role in O157:H7 colonization?

Recent efforts have focused on interactions between O157:H7 and the native bovine microbiota. In cattle, diet strongly shapes the GIT’s microbial composition. Forage-based diets are generally associated with increased prevalence of *Firmicutes* phyla members, whereas high-concentrate diets favor members of the *Bacteroidetes* phylum. Interestingly, attempts to correlate diet, native microbiota, and O157:H7 shedding have been mixed. High-concentrate diets are often associated with altered O157:H7 shedding patterns but shedding also occurs in cattle fed forage-based rations. Additionally, microbial communities found to be positively correlated with O157:H7 are from both *Firmicutes* and *Bacteroidetes*, suggesting complicated microbial dynamics between native GIT populations and O157:H7, regardless of diet [3]. Similar inconsistencies have been observed between non-shedders and super shedders. Taxa such as *Clostridium* and *Bacteroides* have been associated with super shedders [13]; however, other taxa, including *Erysipelotrichaceae* and *Prevotellaceae*, have been associated with both super-shedders and non-shedders, making it difficult to define a consistent microbial signature linked to O157:H7 shedding [3]. A major question remaining is how these microbial populations support O157:H7 persistence at the RAJ. These interactions are hypothesized to involve microbial cross-feeding mechanisms, although the exact pathways remain unclear. Recent work has also raised questions regarding whether native microbial communities influence evolutionary changes in O157:H7, such as the degeneration of its type VI secretion system (T6SS) [14]. Whether evolution of microbial competition systems reflects adaptation to bovine microbial communities or is instead driven by other environmental pressures remains to be determined. Overall, understanding how the native microbiota influences O157:H7 colonization may help identify new pre-harvest strategies that reduce shedding in cattle.

## How does O157:H7 interact with the bovine immune system?

The discovery that bovine vascular endothelial cells lack the Shiga toxin receptor initially led to the assumption that cattle broadly lacked Gb3. However, this assumption was challenged when immune cell populations such as B and T cells were shown to express Gb3 and respond to Shiga toxin exposure [15]. These findings suggested that asymptomatic carriage in cattle may involve suppression or modulation of host immune responses. Further studies demonstrated that although cattle generate Shiga toxin-specific antibodies, toxin binding to B and T lymphocytes hampers lymphocyte proliferation [16]. Additional work has suggested that certain mucosal mesenchymal cell populations also express Gb3 and respond to O157:H7 virulence factors [17]. Although the exact cell type remains unidentified, this suggests antigen-presenting cells may also be targeted, contributing to a dampened adaptive immune response. Gb3 expression has also been identified within bovine ileal crypts. Consistent with this observation, Shiga toxin exposure in a bovine ileal organoid model restricted epithelial proliferation, indicating that altered epithelial homeostasis may represent an additional mechanism promoting persistent colonization [18]. Other immune responses may also influence colonization. Calves experimentally infected with O157:H7 exhibit alterations in the mucin layer and increased mast cell abundance at the RAJ [19]. Whether O157:H7 directly alters mast cell effector functions remains unclear. Several immune signaling pathways are also affected by O157:H7 virulence factors. Effector proteins such as NleE and NleB inhibit NF-kB signaling pathways, reducing proinflammatory responses [20]. O157:H7 has also been shown to downregulate TLR4 signaling, further contributing to impaired NF-kB activation [19]. Taken together, these findings suggest that the ability of O157:H7 to quietly modulate host immune responses may contribute to the variable efficacy observed among vaccine strategies designed to reduce shedding [3]. While many aspects of host immune modulation remain unresolved, a key feature of O157:H7 persistence appears to involve dampening host inflammatory responses and altering epithelial homeostasis to maintain long-term colonization within the bovine GIT.

## Why is understanding O157:H7 colonization important in food safety?

The association between beef products and O157:H7 contamination has driven major changes in food safety practices within processing facilities. Modern post-harvest mitigation strategies utilize a multi-hurdle approach involving both physical (e.g., washing, dehairing) and chemical (e.g., chlorine, organic acids) interventions [6]. These interventions have greatly reduced contamination rates in beef products; however, several challenges with O157:H7 contamination remain. The presence of super shedders in processing plants can place additional strain on post-harvest decontamination strategies by overwhelming critical control points with elevated microbial loads [9]. Considerable work remains in understanding why certain animals become super shedders and developing intervention strategies that can specifically target this subset of cattle. Environmental contamination from fecal shedding is also a major concern. O157:H7 originating from cattle operations can contaminate water sources, fruits, and vegetable crops. Increasingly, outbreaks are associated with fresh produce, where effective post-harvest mitigation is more limited in comparison to beef processing systems [2]. As a result, there is growing interest in developing pre-harvest mitigation strategies that reduce O157:H7 colonization and shedding within live animals. Improved understanding of colonization dynamics may support the development of probiotics, vaccines, bacteriophage therapies, and dietary interventions capable of reducing O157:H7 persistence and shedding. Additionally, understanding which colonization mechanisms are conserved across other clinically important STEC serogroups may facilitate the development of broadly applicable intervention strategies. Ultimately, reducing O157:H7 colonization in cattle remains a key goal for preventing downstream contamination and improving food safety. As foodborne outbreaks linked to O157:H7 continue to occur, understanding how this pathogen colonizes cattle is critical for improving animal management and public health.
